# Inhibition of Macrophage Migration Inhibitory Factor Activity Attenuates Haemorrhagic Shock-Induced Multiple Organ Dysfunction in Rats

**DOI:** 10.3389/fimmu.2022.886421

**Published:** 2022-04-06

**Authors:** Nikita M. Patel, Noriaki Yamada, Filipe R. M. B. Oliveira, Lara Stiehler, Elisabeth Zechendorf, Daniel Hinkelmann, Sandra Kraemer, Christian Stoppe, Massimo Collino, Debora Collotta, Gustavo Ferreira Alves, Hanna Pillmann Ramos, Regina Sordi, Ingo Marzi, Borna Relja, Gernot Marx, Lukas Martin, Christoph Thiemermann

**Affiliations:** ^1^ William Harvey Research Institute, Barts and The London School of Medicine and Dentistry, Queen Mary University of London, London, United Kingdom; ^2^ Gifu University Graduate School of Medicine, Department of Emergency and Disaster Medicine Gifu University Hospital Advanced Critical Care Center, Gifu, Japan; ^3^ Department of Pharmacology, Universidade Federal de Santa Catarina, Florianópolis, Brazil; ^4^ Department of Intensive Care and Intermediate Care, University Hospital RWTH Aachen, Aachen, Germany; ^5^ Department of Anesthesiology & Intensive Care Medicine, University Hospital Würzburg, Würzburg, Germany; ^6^ Department of Neurosciences “Rita Levi Montalcini”, University of Turin, Turin, Italy; ^7^ Department of Trauma, Hand and Reconstructive Surgery, University Hospital Frankfurt, Goethe University, Frankfurt, Germany; ^8^ Experimental Radiology, Department of Radiology and Nuclear Medicine, Otto-von-Guericke University, Magdeburg, Germany

**Keywords:** haemorrhagic shock, ischaemia-reperfusion, ISO-1, macrophage migration inhibitory factor, multiple organ dysfunction syndrome, trauma

## Abstract

**Objective:**

The aim of this study was to investigate (a) macrophage migration inhibitory factor (MIF) levels in polytrauma patients and rats after haemorrhagic shock (HS), (b) the potential of the MIF inhibitor ISO-1 to reduce multiple organ dysfunction syndrome (MODS) in acute (short-term and long-term follow-up) HS rat models and (c) whether treatment with ISO-1 attenuates NF-κB and NLRP3 activation in HS.

**Background:**

The MODS caused by an excessive systemic inflammatory response following trauma is associated with a high morbidity and mortality. MIF is a pleiotropic cytokine which can modulate the inflammatory response, however, its role in trauma is unknown.

**Methods:**

The MIF levels in plasma of polytrauma patients and serum of rats with HS were measured by ELISA. Acute HS rat models were performed to determine the influence of ISO-1 on MODS. The activation of NF-κB and NLRP3 pathways were analysed by western blot in the kidney and liver.

**Results:**

We demonstrated that (a) MIF levels are increased in polytrauma patients on arrival to the emergency room and in rats after HS, (b) HS caused organ injury and/or dysfunction and hypotension (post-resuscitation) in rats, while (c) treatment of HS-rats with ISO-1 attenuated the organ injury and dysfunction in acute HS models and (d) reduced the activation of NF-κB and NLRP3 pathways in the kidney and liver.

**Conclusion:**

Our results point to a role of MIF in the pathophysiology of trauma-induced organ injury and dysfunction and indicate that MIF inhibitors may be used as a potential therapeutic approach for MODS after trauma and/or haemorrhage.

## Introduction

Trauma is one of the leading causes of death and disability in young people aged under 44 and exceeds the number of deaths caused by HIV, tuberculosis and malaria combined (1). Globally, injuries are responsible for over 9% of all mortalities and annually there are approximately 6 million trauma-related deaths (1, 2). Trauma-associated haemorrhage and haemorrhagic shock (HS) account for nearly 40% of all trauma deaths and is a key driver of multiple organ dysfunction (MODS) (3–7).

Whilst the number of early post-injury deaths have decreased in recent years secondary to improved care in the pre-hospital setting, there has been an accompanying increase in deaths attributed to MODS during the late post-injury phase (4, 8, 9). The mechanisms contributing to MODS include (a) an excessive systemic inflammatory response secondary to the release of damage-associated molecular patterns (DAMPs) from extensive tissue damage and (b) ischaemia-reperfusion (I/R) injury (8, 10).

DAMPs activate the immune system, leading to the release of cytokines which can cause organ injury and dysfunction (11). Moreover, raised cytokine levels are linked to worse prognosis in critically ill patients (12–14). One such cytokine is macrophage migration inhibitory factor (MIF) which is pro-inflammatory and possesses chemokine-like properties by promoting the expression or production of several pro-inflammatory mediators including IL-1β, IL-2, IL-6, IL-8, IL-12, IFN-γ, nitric oxide, TNF-α, cyclooxygenase 2 and matrix metalloproteinases (15–23). Consequently, further leukocytes are directed to the site of injury and/or infection (24–26). MIF also has a role in counter-regulating the immunosuppressive and anti-inflammatory effects of glucocorticoids (27, 28).

It has previously been shown that MIF concentration in the plasma/serum of trauma patients was higher than that of healthy controls (29, 30) and serum MIF levels in blunt trauma patients with MODS were significantly greater than patients without MODS (31, 32). Currently, there are no specific pharmacological treatments which prevent the onset of MODS associated with HS. Therefore, the aim of this study was to investigate the effects of blocking MIF activity with the inhibitor ISO-1 [(S,R)-3-(4-hydroxyphenyl)-4,5-dihydro-5-isoxazole acetic acid methyl ester] on the HS-induced MODS in rats.

## Methods

### MIF Gene Expression in Human Whole Blood

Original data was obtained under Gene Expression Omnibus (GEO) accession GSE36809, published by Xiao and colleagues (33). RNA was extracted from whole blood leukocytes of severe blunt trauma patients (n = 167) over the course of 28 days and healthy controls (n = 37) and hybridised onto an HU133 Plus 2.0 GeneChip (Affymetrix) according to the manufacturer’s recommendations. The dataset was reanalysed for MIF gene expression.

### Ethical Statement

Blood samples of 208 patients were collected after written informed consent was obtained from either the patient or a nominated legally authorised representative. Samples were collected between 2010-2014 from University Hospital Frankfurt of Goethe-University and approved by an institutional ethics committee (Number 312/10) in accordance with the declaration of Helsinki and following STROBE-guidelines (34).

For the short-term follow-up acute HS model, all animal procedures were approved by the Animal Welfare Ethics Review Board of Queen Mary University of London and by the Home Office (Licence number PC5F29685). For the long-term follow-up acute HS model, all animal procedures were approved by the Universidade Federal de Santa Catarina Institutional Committee for Animal Use in Research (Licence number 7396250219) in accordance with the Brazilian Government Guidelines for Animal Use in Research. All *in vivo* experiments are reported in accordance to ARRIVE guidelines.

### Patient Study Population and Sample Collection

Blood samples from patients (18-80 years) with blunt or penetrating trauma and ISS ≥ 16 were obtained on Day 0 (arrival to the emergency room); Day 2; Day 5 and Day 7. Exclusion criteria were patient death in the emergency room or within 24 h of hospital admission, known pre-existing immunological disorders, treatment with immunosuppressive or anti-coagulant medication, burns, concomitant acute myocardial infarction and thromboembolic events. Blood samples were collected in pre-chilled ethylenediaminetetraacetic acid tubes (BD vacutainer, Becton Dickinson Diagnostics, Aalst, Belgium) and kept on ice. Blood was centrifuged at 2,000 g for 15 min at 4°C to separate serum and stored at −80°C for further analysis.

### Experimental Design

Male Wistar rats (for short-term follow-up acute model: Charles River Laboratories Ltd., UK; for long-term follow-up acute model: Universidade Federal de Santa Catarina, Brazil) weighing 250-350 g were kept under standard laboratory conditions and received a chow diet and water *ad libitum*. ISO-1 (25 mg/kg; Tocris, UK) was diluted in 5% DMSO + 95% Ringer’s Lactate (vehicle) and rats were treated (i.v. in short-term follow-up and i.p. in long-term follow-up) upon resuscitation.

### Acute Haemorrhagic Shock Model (Short-Term Follow-Up)

The pressure-controlled short-term follow-up acute HS model was performed as previously described (35–38). Briefly, forty rats were anaesthetised with sodium thiopentone (120 mg/kg i.p. initially and 10 mg/kg i.v. for maintenance as needed and randomised into four groups (n = 10 per group): Sham + vehicle; Sham + ISO-1 (25 mg/kg), HS + vehicle; HS + ISO-1 (25 mg/kg) using the GraphPad online random number generator. The investigator was blinded to the intervention (vehicle or ISO-1) and treatment group allocation was revealed following data analysis. Analgesia was not administered as the rats remain anaesthetised for the duration of the experiment (non-recovery procedure) and as such do not expect the animals to feel pain. Adequacy of anaesthesia was ascertained throughout the experiment by testing the pedal reflex. No animals died during the course of the study, thus all data have been included. Blood was withdrawn to achieve a fall in mean arterial pressure (MAP) to 35 ± 5 mmHg, which was maintained for 90 min. At 90 min after initiation of haemorrhage (or when 25% of the shed blood had to be reinjected to sustain MAP at 35 ± 5 mmHg), resuscitation was performed with the shed blood over a period of 5  min. At 4 h post-resuscitation, blood was collected for the measurement of biomarkers of organ injury/dysfunction (MRC Harwell Institute, Oxfordshire, UK) and organs for *ex vivo* analysis. Sham-operated rats were used as control and underwent identical surgical procedures, but without haemorrhage or resuscitation. Detailed description of the short-term follow-up model can be found in the supplemental (**Supplemental Figure 1A**).

### Acute Haemorrhagic Shock Model (Long-Term Follow-Up)

The pressure-controlled long-term follow-up acute HS model was performed as previously described (36). Briefly, thirty rats were administered analgesia with tramadol (10 mg/kg i.p.) 15 min prior to anaesthesia induction with ketamine and xylazine (100 mg/kg and 10 mg/kg i.m. respectively) and randomised into three groups: Sham + vehicle (n = 6); HS + vehicle (n = 12); HS + ISO-1 (25 mg/kg; n = 12) using the GraphPad online random number generator. The investigator was blinded to the intervention (vehicle or ISO-1) and treatment group allocation was revealed following data analysis. Adequacy of anaesthesia throughout the experiment was ascertained by testing the pedal reflex. No animals died during the course of the study, thus all data have been included. Blood was withdrawn to achieve a fall in MAP to 40 ± 2 mmHg, which was maintained for 90 min. At 90 min after initiation of haemorrhage (or when 25% of the shed blood had to be reinjected to sustain MAP at 40 ± 2 mmHg), resuscitation was performed with the shed blood over a period of 5 min plus 1.5 mL/kg Ringer’s lactate. At 24 h post-resuscitation, blood was collected for the measurement of organ injury/dysfunction parameters (Hospital Universitário Professor Polydoro Ernani de São Thiago, Brazil) and organs for *ex vivo* analysis. Sham-operated rats were used as control and underwent identical surgical procedures, but without haemorrhage or resuscitation. Detailed description of the long-term follow-up model can be found in the supplemental (**Supplemental Figure 1B**).

### MIF ELISA

Human MIF plasma levels (R&D SYSTEMS Human MIF DuoSet) and rat MIF serum levels from the acute HS (short-term follow-up) model (Cusabio Biotech, Wuhan, China) were detected by commercially available ELISAs according to the manufacturer protocol. Detection occurred at 450 nm and 540 nm using iMark^®^ microplate absorbance reader (BioRad). Further details can be found in the **Supplemental Table 1**.

### Western Blot Analysis

Semi-quantitative immunoblot analysis was carried out in kidney and liver samples as previously described (36). Detailed description of the method can be found in the supplemental.

### CD68 Immunohistochemical Staining

Lung tissue sections were deparaffinised and hydrated and stained for CD68. Detailed description of the method can be found in the supplemental.

### Quantification of Myeloperoxidase Activity

Determination of myeloperoxidase activity in lung and liver tissue samples was performed as previously described (36). Detailed description of the method can be found in the supplemental.

### Statistical Analysis

All figures are expressed as median with range of *n* observations, where *n* represents the number of animals/experiments/subjects studied. Measurements obtained from the vehicle and ISO-1 treated animal groups were analysed by one-way ANOVA followed by a Bonferroni’s *post-hoc* test on GraphPad Prism 8.0 (GraphPad Software, Inc., La Jolla, CA, USA). The distribution of the data was verified by Shapiro-Wilk normality test, and the homogeneity of variances by Bartlett test. When necessary, values were transformed into logarithmic values to achieve normality and homogeneity of variances. To investigate the relationship between the variables, Pearson correlation *r* was performed. P<0.05 was considered statistically significant.

## Results

### MIF Gene Expression Is Elevated in Trauma Patients

Xiao and colleagues (33) compared genome-wide expression in leukocytes from trauma patients against matched healthy controls. We reanalysed this dataset for MIF expression. When compared to healthy controls, MIF expression was significantly elevated at all time points except Day 1 (p<0.05; **Supplemental Figure 2**). An initial increase was noted at 12 h followed by a later peak at Day 7. MIF expression remained elevated at Day 28, the latest timepoint measured.

### MIF Gene Expression Does Not Differ Between Uncomplicated and Complicated Recovery Patient Groups

Xiao and colleagues (33) also stratified their trauma patient cohort into uncomplicated (recovery in <5 days) and complicated (recovery after 14 days, no recovery by Day 28 or death) to further identify genotypic differences. We reanalysed this dataset for MIF expression using this stratification. When comparing uncomplicated and complicated patients, there were no significant differences at any of the timepoints measured (p>0.05; **Supplemental Figure 3**).

### Plasma MIF Levels Are Elevated in Polytrauma Patients and Associated With Longer Stay in ICU and Hospital

**Figure 1:**
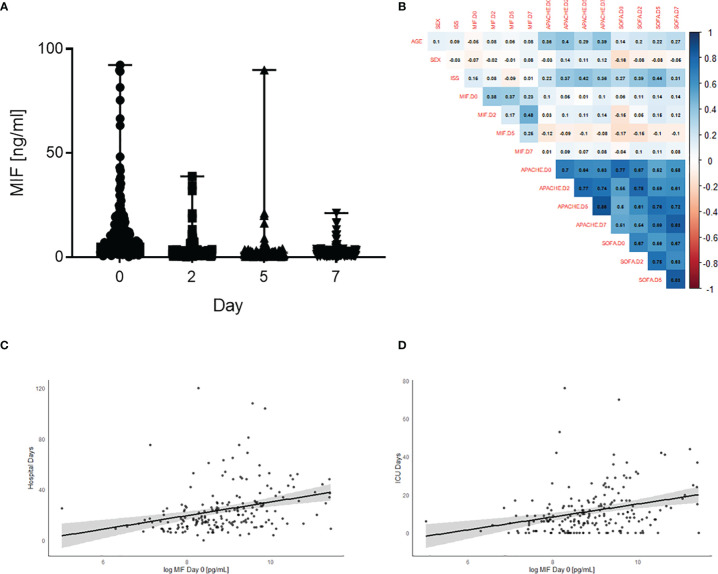
Plasma MIF levels are elevated in polytrauma patients and associated with longer stay in ICU and hospital. **(A)** Plasma MIF levels in trauma patients (n = 208) at different time points: Day 0 (Emergency room), Day 2, Day 5 and Day 7. Data are expressed as median with range. **(B)** Heatmap for correlations between MIF levels and baseline characteristics sex, age, SOFA, ISS and APACHE score. Scatter plot for Day 0 MIF levels against **(C)** hospital stay and **(D)** intensive care unit (ICU) stay.

To investigate the role of MIF in trauma, 208 patients were included in the study. Detailed patient characteristics can be found in **Table 1**. Polytrauma patients showed significantly increased MIF levels on Day 0 (on arrival to the emergency room, 12398 ± 1262 pg/mL) compared to Day 2 (2866.9 ± 377.8 pg/mL), Day 5 (2335.7 ± 203.4 pg/mL) and Day 7 (2114.6 ± 165.3 pg/mL) (all p<0.001; **Figure 1A**). Furthermore, we found a weak positive correlation between MIF levels on Day 0 and both hospital (r = 0.22, n = 199, p<0.01; **Figure 1C**) and ICU (r = 0.26, n = 198, p<0.01; **Figure 1D**) stays. MIF levels on Day 0 were not correlated with baseline characteristics such as age (r = -0.05, n = 200, p = 0.49) and sex (r = -0.07, n = 200, p = 0.3) and only weakly correlated with ISS score (r = 0.15, n = 177, p<0.05) (**Figure 1B**).

**Table 1:** Trauma patient clinical characteristics.

	Trauma (n = 208)
Age (year) (IQR)	47.0 (31-60)
Male sex (%)	156 (75.0)
SOFA (points) (IQR)	5.00 (1.0-7.0)
APACHE II (points) (IQR)	16.0 (6.0-22.0)
ISS score (points) (IQR)	23.0 (17.0-32.0)
LOS ICU (days) (IQR)	8.0 (4.0-15.0)
LOS In-hospital (days) (IQR)	19.0 (13.0-29.0)
MIF D0 [pg/ml] (IQR)	6839 (3713-14205)
MIF D2 [pg/ml] (IQR)	1598.0 (1080.0-2555.5)
MIF D5 [pg/ml] (IQR)	1137.0 (650.0-1960.0)
MIF D7 [pg/ml] (IQR)	1331.9 (814.2-2158.5)

Data are presented as n (%) or median (IQR). D0/2/5/7: Day 0/2/5/7; ICU, intensive care unit; IQR, interquartile ranges (Q1-Q3); LOS, length of stay.

### MIF Levels Are Elevated in Serum of Rats After Induction of Acute HS (Short-Term Follow-Up)

**Figure 2:**
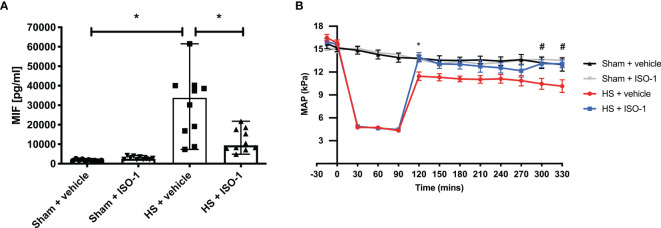
Serum MIF levels are elevated in HS-rats and ISO-1 improves HS-induced circulatory failure in a short-term follow-up acute HS model. **(A)** Serum MIF levels were detected by ELISA in vehicle or ISO-1 treated rats. Data are expressed as median with range of ten animals per group. **(B)** Mean arterial pressure (MAP) was measured from the completion of surgery to the termination of the experiment for all groups. Statistical analysis was performed using one-way ANOVA followed by a Bonferroni’s *post-hoc* test. ^*^p < 0.05 Sham + vehicle vs. HS + vehicle; ^#^p < 0.05 HS + vehicle vs. HS + ISO-1.

Having found elevated plasma MIF levels in patients with trauma-haemorrhage, we investigated whether haemorrhage alone (in the absence of physical trauma) is sufficient to drive increases in MIF. To address this question, we used a model of severe haemorrhage followed by resuscitation in the rat. When compared to sham-operated rats, haemorrhage followed by resuscitation resulted in a significant increase in MIF levels (p<0.001; **Figure 2A**). Although ISO-1 has been reported to inhibit the effects, rather than the formation, of MIF *in vivo*, we report here that treatment of HS rats with ISO-1 resulted in a significantly lower MIF level when compared to HS rats treated with vehicle (p<0.05; **Figure 2A**). Administration of ISO-1 to sham-operated rats had no effect on MIF levels (p>0.05; **Figure 2A**).

### Treatment With ISO-1 Improves HS-Induced Circulatory Failure in Acute HS (Short-Term Follow-Up)

To investigate the effects of ISO-1 on circulatory failure, MAP was measured from the completion of surgery to the termination of the experiment. Baseline MAP values were similar amongst all four groups. Rats subjected to HS demonstrated a decline in MAP which was ameliorated by resuscitation, but still remained lower than that of sham-operated rats during resuscitation (at the equivalent time points, **Figure 2B**). When compared to sham-operated rats, HS-rats treated with vehicle exhibited a more pronounced decrease in MAP over time post-resuscitation. In contrast, MAP of HS-rats treated with ISO-1 was significantly higher than HS-rats treated with vehicle 4 h post-resuscitation (p<0.001; **Figure 2B**).

### Treatment With ISO-1 Attenuates HS-Induced Organ Damage in Acute HS (Short-Term Follow-Up)

**Figure 3:**
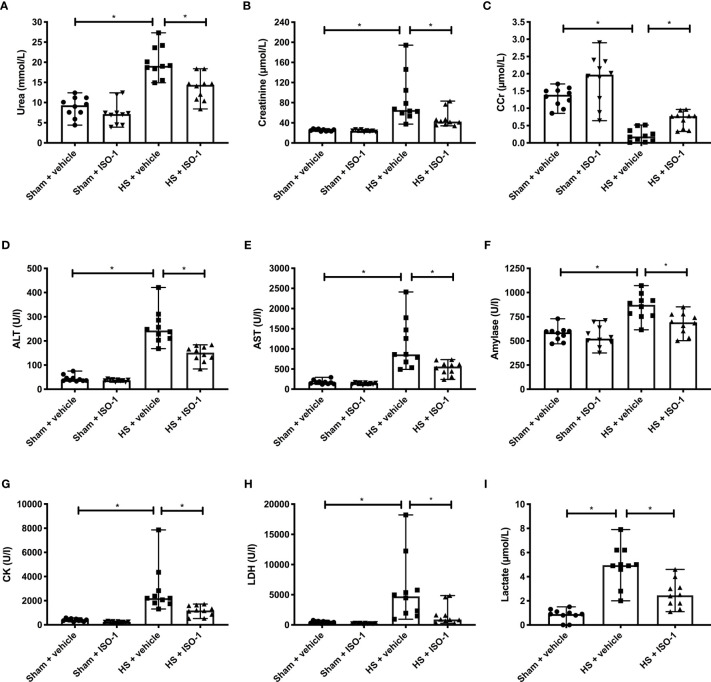
Treatment with ISO-1 attenuates HS-induced organ damage in a short-term follow-up acute HS model. Rats were subjected to haemorrhagic shock (HS) and 4 h after resuscitation, levels of serum **(A)** urea, **(B)** creatinine, **(C)** creatinine clearance (CCr), **(D)** alanine aminotransferase (ALT), **(E)** aspartate aminotransferase (AST), **(F)** amylase, **(G)** creatine kinase (CK), **(H)** lactate dehydrogenase (LDH) and **(I)** lactate were determined. Sham-operated rats were used as control. Data are expressed as median with range of ten animals per group. Statistical analysis was performed using one-way ANOVA followed by a Bonferroni’s *post-hoc* test. ^*^p < 0.05 denoted statistical significance.

Having demonstrated that treatment with ISO-1 improves HS-induced circulatory failure, we next explored whether ISO-1 attenuates MODS associated with HS in rats. When compared to sham-operated rats, rats subjected to HS and treated with vehicle displayed increases in serum urea (p<0.001; **Figure 3A**) and creatinine (p<0.001; **Figure 3B**) and a decrease in creatinine clearance (p<0.001; **Figure 3C**) indicating the development of renal dysfunction. When compared to sham-operated rats, vehicle treated HS-rats exhibited significant increases in both ALT (p<0.001; **Figure 3D**) and AST (p<0.001; **Figure 3E**) indicating the development of hepatic injury, while the increases in amylase (p<0.001; **Figure 3F**) and CK (p<0.001; **Figure 3G**) denote pancreatic and neuromuscular injury, respectively. The significant increase in LDH (p<0.001; **Figure 3H**) in HS-rats treated with vehicle confirmed tissue injury whilst the increase in lactate (p<0.001; **Figure 3I**) indicated decreased transport of oxygen to the tissues developing from the state of hypoperfusion. Treatment of HS-rats with ISO-1 significantly attenuated the renal dysfunction, hepatic injury, pancreatic injury, neuromuscular injury and general tissue damage caused by HS (all p<0.05; **Figures 3A–I**). As HS causes macrophage infiltration into the lungs, we measured CD68^+^ positive cells as a marker for macrophage invasion. When compared to sham-operated rats (21.75 ± 2.29 per field), HS-rats treated with vehicle displayed a significant increase in macrophage count (47.33 ± 8.75 per field, p<0.05). Treatment with ISO-1 in HS-rats did not result in a significant decrease in macrophage count (35.90 ± 4.30 per field; **Supplemental Figure 4**).

### Serum MIF Levels Are Strongly Associated With Clinical Chemistry and MAP in Acute HS (Short-Term Follow-Up)

Having shown rats subjected to HS had elevated serum MIF levels, we wished to elucidate whether this observed increase correlates with clinical chemistry parameters (measured in serum collected 4 h post-resuscitation) and MAP (measured at 4 h post-resuscitation before sample collection). There was a positive correlation between MIF and all clinical chemistry parameters (p<0.05; **Supplemental Figure 5** shown in blue, r values range from 0.64 – 0.77), with the strongest correlations between AST (r = 0.77) and LDH (r = 0.72). There was a negative correlation between MIF and MAP (p<0.05; **Supplemental Figure 5** shown in red, r = -0.66).

### Treatment With ISO-1 Attenuates Hepatic and Renal NF-κB Activation in Acute HS (Short-Term Follow-Up)

**Figure 4:**
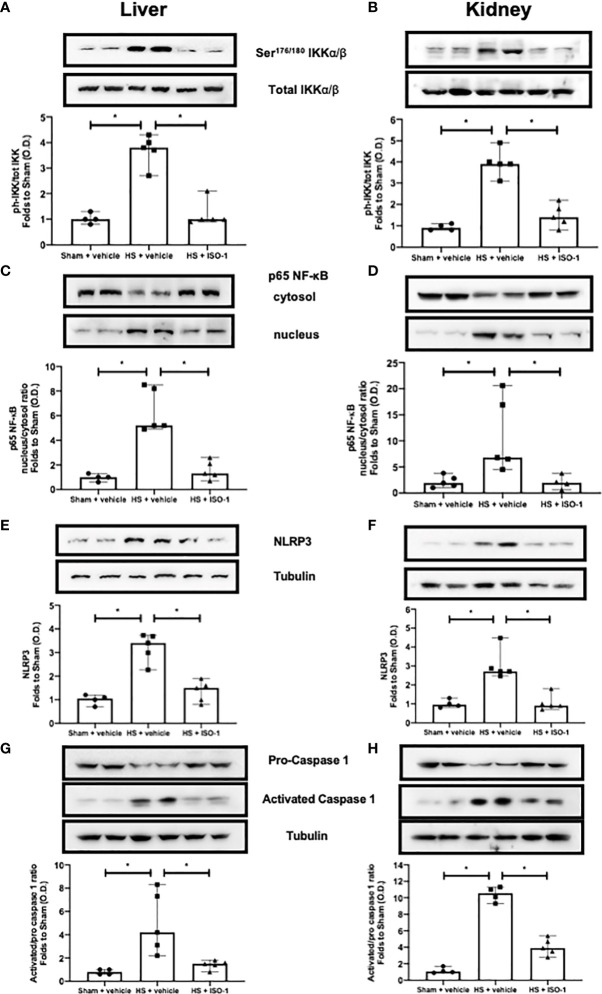
Treatment with ISO-1 attenuates NF-κB and NLRP3 activation in a short-term follow-up acute HS model. **(A, B)** The phosphorylation of IKKα/β at Ser^176/180^, **(C, D)** nuclear translocation of p65, **(E, F)** activation of NLRP3 and **(G, H)** cleavage of pro-caspase 1 of vehicle and ISO-1 treated rats were determined by western blot in the liver and kidney. Protein expression was measured as relative optical density (O.D.) and normalised to the sham band. Data are expressed as median with range of 4-5 animals per group. Statistical analysis was performed using one-way ANOVA followed by a Bonferroni’s *post-hoc* test. *p < 0.05 denoted statistical significance.

The effect of MIF inhibition on the activation of the signalling events leading to the activation of NF-κB, was investigated in the kidney and liver. When compared to sham-operated rats, HS-rats treated with vehicle had significant increases in the phosphorylation of IKKα/β at Ser^176/180^ (p<0.001; **Figure 4A** and p<0.001; **Figure 4B**) and translocation of p65 to the nucleus (p<0.001; **Figure 4C** and p<0.05; **Figure 4D**). Treatment with ISO-1 significantly attenuated the increases in hepatic and renal phosphorylation of IKKα/β at Ser^176/180^ (p<0.001; **Figure 4A** and p<0.001; **Figure 4B**) and the nuclear translocation of p65 (p<0.001; **Figure 4C** and p<0.05; **Figure 4D**).

### Treatment With ISO-1 Attenuates Hepatic and Renal NLRP3 Inflammasome Activation in Acute HS (Short-Term Follow-Up)

Having discovered treatment with ISO-1 significantly reduced the activation of NF-κB in the kidney and liver of rats subjected to HS, we next analysed the potential involvement of the NLRP3 inflammasome. When compared to sham-operated rats, HS-rats treated with vehicle exhibited a significantly increased expression of the NLRP3 inflammasome (p<0.001; **Figure 4E** and p<0.001; **Figure 4F**) and cleavage of pro-caspase 1 to caspase 1 (p=0.008; **Figure 4G** and p<0.001; **Figure 4H**). Treatment with ISO-1 significantly inhibited the hepatic and renal expression of NLRP3 (p<0.001; **Figure 4E** and p<0.001; **Figure 4F**) and cleavage of pro-caspase 1 to caspase 1 (p=0.015; **Figure 4G** and p<0.001; **Figure 4H**).

### Treatment With ISO-1 Improves HS-Induced Circulatory Failure in Acute HS (Long-Term Follow-Up)

**Figure 5:**
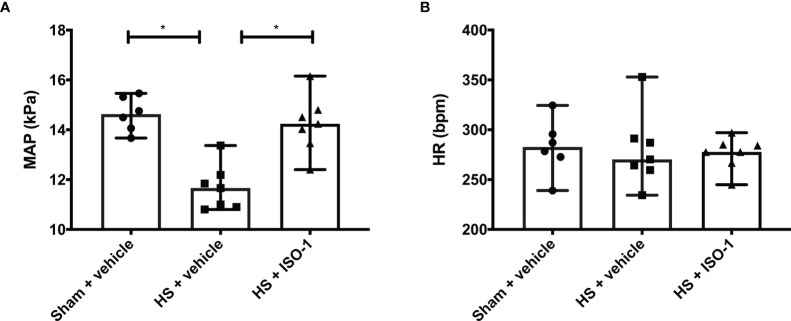
Treatment with ISO-1 improves HS-induced cardiac dysfunction in a long-term follow-up acute HS model. **(A)** Mean arterial pressure (MAP) and **(B)** heart rate (HR) were measured 24 h post resuscitation for vehicle and ISO-1 treated rats. Data are expressed as median with range. Sham + vehicle (n = 6), HS + vehicle (n = 7) and HS + ISO-1 (n = 7). Statistical analysis was performed using one-way ANOVA followed by a Bonferroni’s *post-hoc* test. *p < 0.05 denoted statistical significance.

Having demonstrated treatment with ISO-1 improved blood pressure in a short-term follow-up model, we wished to determine whether ISO-1 would still be effective in a model in which the resuscitation period is prolonged to 24 h. When compared to sham-operated rats, HS-rats treated with vehicle had significantly lower MAP values at 24 h post-resuscitation (p<0.001; **Figure 5A**); highlighting that either cardiovascular dysfunction or excessive hypotension was still present. In contrast, MAP of HS-rats treated with ISO-1 was significantly higher at 24 h than vehicle treated rats (p<0.05; **Figure 5A**). There were no significant differences in HR between any of the three groups investigated (p>0.05; **Figure 5B**).

### Treatment With ISO-1 Attenuates HS-Induced Organ Damage and Myeloperoxidase Activity in Acute HS (Long-Term Follow-Up)

**Figure 6:**
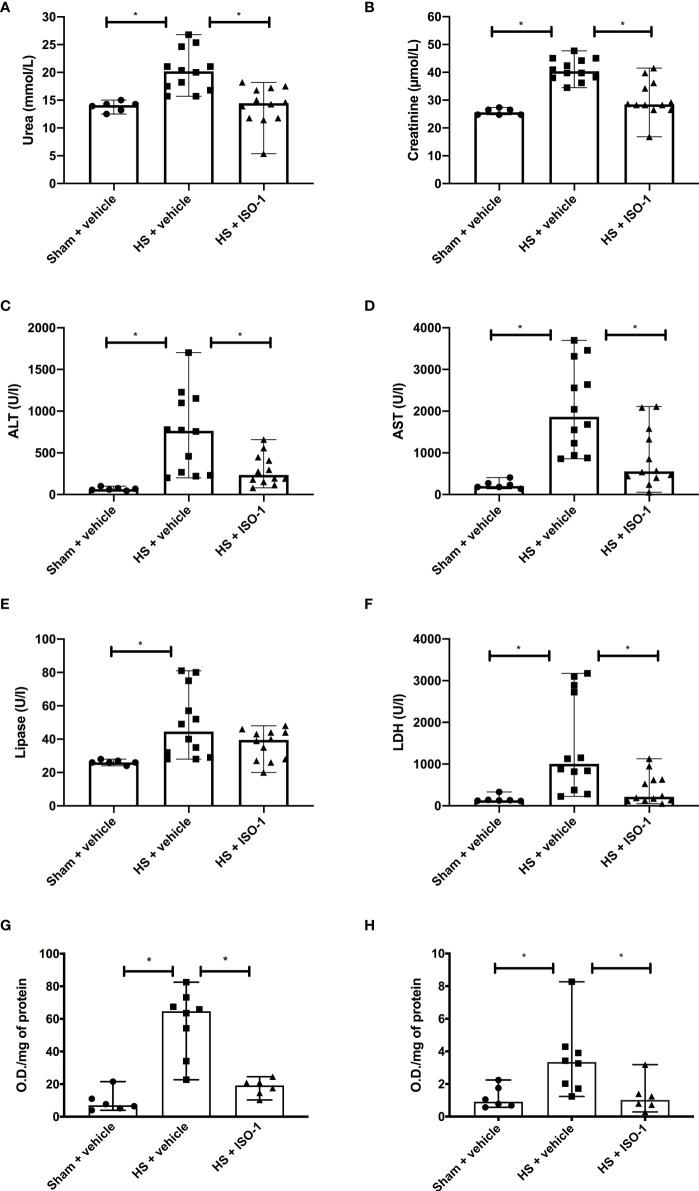
Treatment with ISO-1 attenuates HS-induced organ damage and myeloperoxidase activity in a long-term follow-up acute HS model. Rats were subjected to haemorrhagic shock (HS) and 24 h after resuscitation, levels of serum **(A)** urea, **(B)** creatinine, **(C)** alanine aminotransferase, (ALT), **(D)** aspartate aminotransferase (AST), **(E)** lipase and **(F)** LDH were determined were determined in vehicle and ISO-1 treated rats. Sham-operated rats were used as control. Sham + vehicle (n = 6), HS + vehicle (n = 12) and HS + ISO-1 (n = 12). Myeloperoxidase activity in **(G)** lung and **(H)** liver were determined for vehicle and ISO-1 treated rats. Sham + vehicle (n = 6), HS + vehicle (n = 8) and HS + ISO-1 (n = 6). Data are expressed as median with range. Statistical analysis was performed using one-way ANOVA followed by a Bonferroni’s *post-hoc* test. *p<0.05 denoted statistical significance.

Having shown that treatment with ISO-1 ameliorated the MODS associated with HS in a short-term follow-up model, we examined whether this effect was sustained when the resuscitation period was extended to 24 h. As with the short-term follow-up model, when compared to sham-operated rats, rats subjected to HS with long-term follow-up and treated with vehicle displayed significant increases in serum urea (p<0.05; **Figure 6A**) and creatinine (p<0.001; **Figure 6B**) indicating the development of renal dysfunction. When compared to sham-operated rats, vehicle treated HS-rats exhibited significant increases in ALT (p<0.001; **Figure 6C**), AST (p<0.001; **Figure 6D**), lipase (p<0.05; **Figure 6E**) and LDH (p<0.05; **Figure 6F**). Treatment of HS-rats with ISO-1 significantly attenuated the renal dysfunction, hepatic injury and tissue damage caused by HS (all p<0.05; **Figures 6A–D, F**). Having demonstrated that treatment with ISO-1 reduced the cell infiltration in the lung in a short-term follow-up acute HS model, we measured myeloperoxidase (MPO) activity in the lung and liver as an indicator of neutrophil infiltration. When compared to sham-operated rats, HS-rats treated with vehicle showed a significant increase in MPO activity in the lung (p<0.001; **Figure 6G**) and liver (p<0.05; **Figure 6H**). Treatment with ISO-1 in HS-rats significantly attenuated these rises in MPO activity (p<0.001; **Figure 6G** and p<0.05; **Figure 6H**).

## Discussion

This study reports that inhibition of MIF activity attenuates organ injury/dysfunction and circulatory failure in acute short-term follow-up (**Figures 2**, **3**) and long-term follow-up (**Figures 5**, **6**) rat models of HS. Having shown that MIF gene expression is significantly elevated in leukocytes of trauma patients **(Supplemental Figure 2**) and plasma MIF levels are raised in polytrauma patients (**Figure 1**), we used a reverse translational approach to investigate whether pharmacological intervention with ISO-1 ameliorates the MODS associated with HS in a well-established rat model. Inhibition of MIF activity significantly attenuated the fall in blood pressure (**Figure 2** short-term follow-up and **Figure 5** long-term follow-up) and, hence, the delayed vascular decompensation caused by HS (39). Moreover, ISO-1 significantly attenuated the renal dysfunction, hepatic injury and neuromuscular injury caused by HS (**Figure 3** short-term follow-up and **Figure 6** long-term follow-up); highlighting the drug efficacy at both timepoints. Similarly, ISO-1 also reduces disease severity in animal models of sepsis (40), acute pancreatitis (41–46), pneumonia (47), asthma (48), COPD (49), cystitis (50) and colitis (51).

We have illustrated that severe haemorrhage followed by resuscitation in the rat caused a significant increase in serum MIF levels, the magnitude of which was similar to the one seen in trauma patients. This finding implies that haemorrhage (rather than physical trauma) is the main driver for the observed increase in MIF in rats and possibly also in humans. Whilst the elevated plasma MIF levels in our cohort of polytrauma patients do not necessarily indicate an increase in synthesis, the increased gene expression measured by Xiao and colleagues (33) does suggest an associated rise in MIF production.

Post-traumatic complications can result in prolonged stays in ICU and in hospital. In the acute setting, there is a lack of diagnostic or risk stratification tools which allow the identification of the potential clinical outcome of trauma patients. We determined a significant positive correlation between elevated MIF levels in polytrauma patients and the overall length of ICU and hospital stays. These findings indicate that high plasma MIF levels at time of hospital admission are strongly predictive for a longer stay in ICU and hospital. Indeed, the human plasma MIF levels and gene expression data provide evidence supporting the role of MIF in trauma, as both gene expression and plasma levels increase following trauma. Most notably, MIF was a better predictor of hospital stay than either ISS or SOFA scores. For every further increase of MIF on admission by 5000 pg/mL, the stay of patients in hospital is prolonged by ~1.2 days. Similarly, Cho and colleagues showed that trauma patients with elevated MIF levels had longer ICU stays than patients with lower or normal MIF levels (52).

Nevertheless, we found a weak positive but significant correlation between MIF levels on admission and ISS which supports the findings of Chuang and colleagues’ study illustrating higher MIF levels were associated with worse clinical severity scores (APACHE II, RTS and TRISS) (30). Although clinical severity scores cannot be measured in rats, we found significant positive correlations between serum MIF levels and clinical chemistry parameters and a significant negative correlation between MIF and MAP both of which can be considered as indicators organ function (**Supplementary Figure 5**).

What, then, are the mechanisms by which ISO-1 attenuates HS-associated organ injury/dysfunction? It is recognised that key signalling pathways, such as those leading to NF-κB activation, initiate the production of pro-inflammatory mediators such as cytokines, chemokines and enzymes (53). As part of a positive feedback mechanism, these inflammatory mediators can induce activation of NF-κB and its upstream signalling machinery, further amplifying and propagating the NF-κB-mediated inflammatory responses. This can result in a more permeable endothelium, hypoxic/hypoperfused tissues, tissue injury and ultimately MODS (54). Trauma has been shown to increase the translocation of NF-κB to the nucleus (35–38). Inhibition of MIF activity with ISO-1 reduced NF-κB activation in the kidney and liver of HS-rats (**Figure 4**). This may suggest that inhibiting NF-κB activation contributes to the observed protective effects of ISO-1 in HS. It should be noted that MIF has been shown to upregulate TLR4 expression, leading to increased translocation of NF-κB into the nucleus (55) and interact with thioredoxin-interacting protein to induce NF-κB activity (56).

Activation of the NLRP3 inflammasome stimulates IL-1β production which plays a crucial role in trauma-associated systemic inflammation and organ dysfunction/injury (36). Inhibition of MIF activity with ISO-1 reduced both the assembly and subsequent activation of the NLRP3 inflammasome in the kidney and liver of HS-rats (**Figure 4**). This may suggest that inhibiting NLRP3 inflammasome activation contributes to the observed beneficial effects of ISO-1 in HS by decreasing the pro-inflammatory effects related to increased IL-1β production and ensuing tissue inflammation (57). Of note, MIF has been proposed to play a role in the activation of the NLRP3 inflammasome (58, 59).

The sterile inflammation caused by HS is associated with increased recruitment of leukocytes to the tissues and is secondary to NF-κB and NLRP3 activation and their transcriptional regulation of pro-inflammatory cytokines (60–62). Furthermore, the leukocyte and endothelial cell surface expression of adhesion molecules is regulated by NF-κB and promotes leukocyte extravasation from the circulation to the injury site (63). We found a significant increase in CD68^+^ cells in the lung (**Supplementary Figure 4**) and in pulmonary and hepatic MPO activity (**Figure 6**), markers of macrophage and neutrophil recruitment respectively, after induction of HS in rats. Treatment of HS-rats with ISO-1 did not significantly reduce the number of pulmonary CD68^+^ cells and thus, macrophage infiltration into the lung. In contrast, administration of ISO-1 to HS-rats attenuated the rise in MPO activity related to increased neutrophil recruitment. Taken together, these results could imply that ISO-1 attenuates neutrophil, but not macrophage recruitment in HS. Indeed, an anti-MIF antibody was shown to attenuate the LPS-induced migration and accumulation of neutrophils in the lung (64). These observations can be explained by the chemokine-like properties of MIF, which facilitate the activation and recruitment of leukocytes during immune surveillance and inflammation (65, 66). Initially, MIF was eponymously described by its ability to inhibit random macrophage migration *in vitro* (67, 68). However, it is now known that MIF can mediate the recruitment of mononuclear cells in a number of disease states (69–72). A possible explanation as to why ISO-1 did not significantly reduce pulmonary macrophage invasion is that the presence of other inflammatory mediators, such as IL-1β, IL-6 and TNF-α, could have stimulated the migration of macrophages, but we would need to measure these mediators to confirm this theory.

## Limitations

Although ISO-1 displayed some striking, beneficial effects in the acute HS models, there are study limitations which should be taken into consideration. In total, 208 patients were included in the study, however, in some instances there were insufficient plasma volumes available for some of the measured timepoints. Therefore, some correlation analyses were performed with a reduced number of samples/patients. Following trauma, there is an increased inflammatory response, and we were able to measure elevated plasma MIF levels in polytrauma patients. However, we cannot exclude the possibility that the increased plasma levels are not exclusively associated with polytrauma. The animal models used in our study do not encompass all aspects of trauma/HS and further long-term survival experiments are needed to verify that the observed early reduction in MODS does, indeed, translate to improved outcome and ultimately reduced mortality. Hence, caution must be exercised when interpreting the pre-clinical results and extrapolating to the clinical scenario. Additionally, future studies in larger animals/higher species may be useful to confirm efficacy and to further examine the mechanism(s) of action (e.g. microcirculatory effects and blood gas analysis) of ISO-1 in HS. It should be noted that only healthy young male rats were used and, hence, age and gender differences and the presence of co-morbidities were not investigated (but may well impact outcome). Moreover, clinical studies with larger cohorts of trauma patients are required to robustly examine the relationship between MIF activity, inhibition and clinical outcomes in humans. Whilst we did not measure the long-term stability of MIF in the patient samples collected between 2010-2014, we were able to demonstrate significant and time-related elevations in MIF in the blood of patients with trauma and haemorrhage. Indeed, the peak levels of MIF observed in patients on admission to the hospital (measured in samples that were several years old) were similar to the peak levels measured in rats with severe haemorrhage (that we measured within weeks of completing the experiments). Nevertheless, we cannot, however, entirely rule out that the levels of MIF in patients with trauma-haemorrhage would have been even higher if the MIF determinations would have been carried out earlier after the trauma occurred.

## Conclusions

In conclusion, we demonstrate here for the first time that MIF levels are elevated in polytrauma patients on arrival to the emergency room and higher MIF levels are associated with longer stays in ICU and hospital overall. The finding that HS alone (in the absence of physical trauma) in rats resulted in a rise in MIF levels similar to that seen in polytrauma patients supports the view that haemorrhage is the main driver for the elevations in MIF. Furthermore, treatment with ISO-1 reduces the organ injury/dysfunction and circulatory failure caused by severe haemorrhage in the rat, highlighting a role of MIF in disease pathogenesis. Administration of ISO-1 attenuates the degree of NF-κB and NLRP3 inflammasome activation (measured in the kidney and liver), both of which are key drivers of local and systemic inflammation. Thus, we propose that MIF inhibitors may be used in trauma patients to lower the organ injury and inflammation caused by severe haemorrhage and resuscitation.

## Data Availability Statement

Publicly available datasets were analysed in this study. This data can be found here: https://www.ncbi.nlm.nih.gov/geo/query/acc.cgi?acc=gse36809.

## Ethics Statement

The studies involving human participants were reviewed and approved by University Hospital Frankfurt of Goethe-University - Institutional Ethics Committee (Number 312/10). The patients/participants provided their written informed consent to participate in this study. The animal study was reviewed and approved by Animal Welfare Ethics Review Board of Queen Mary University of London and by the Home Office (Licence number PC5F29685) and Universidade Federal de Santa Catarina Institutional Committee for Animal Use in Research (Licence number 7396250219).

## Author Contributions

Conception and design: NMP, LM, and CT. Animal experiments: NY, NMP, FRMBO, HPR, and RS. Human sample analysis: LM, EZ, GM, IM, BR, DH, LS, and CS. Animal sample analyses: NY, NMP, LM, CT, LS, EZ, FRMBO, HPR, SK, DC, MC, GFA, and RS. Clinical study and patient data analyses: LM, EZ, CT, IM, BR, DH, LS, NMP, and CS. Statistical analyses: NY, NMP, DH, LM, and CT. Drafting the manuscript for important intellectual content: LS, LM, EZ, NMP, and CT. All authors reviewed and approved the manuscript.

## Funding

NMP was funded by the William Harvey Research Foundation. HPR and FRMBO were funded by National Council for Scientific and Technological Development (CNPq) fellowship. This study was supported by the German Research Foundation to LM (DFG, MA 7082/3-1), to CS (DFG, STO 1099/8-1) and by an intramural grant to EZ (START 131/19), National Council for Scientific and Technological Development to RS (CNPq, Brazil, Grant 409018/2018-0).

## Conflict of Interest

The authors declare that the research was conducted in the absence of any commercial or financial relationships that could be construed as a potential conflict of interest.

## Publisher’s Note

All claims expressed in this article are solely those of the authors and do not necessarily represent those of their affiliated organizations, or those of the publisher, the editors and the reviewers. Any product that may be evaluated in this article, or claim that may be made by its manufacturer, is not guaranteed or endorsed by the publisher.
