# Supplementary material for: Inhibition of Macrophage Migration Inhibitory Factor Activity Attenuates Haemorrhagic Shock-Induced Multiple Organ Dysfunction in Rats
Source: Front Immunol. 2022 Apr 6;13:886421. doi: 10.3389/fimmu.2022.886421 (PMC9019168; doi:10.3389/fimmu.2022.886421)
Supplement: Supplementary file 1 [file DataSheet_1.pdf]

Supplementary Material

Supplementary Methods

Acute Haemorrhagic Shock Model (Short-term Follow-up)

Rats were anaesthetised with sodium thiopentone (120 mg/kg i.p. initially and 10 mg/kg i.v. for maintenance as needed) and the pedal reflex was tested to ensure adequate anaesthesia. Cannulation with polyethylene catheters (Smiths Medical International Ltd., Kent, UK) of the trachea for facilitation of spontaneous breathing (internal diameter [ID] 1.67 mm), left femoral artery for recording of the mean arterial pressure (MAP) (ID 0.40 mm), left carotid artery for blood withdrawal (ID 0.58 mm) and right jugular vein for fluid and drug administration (ID 0.40 mm) was performed. To prevent tissue desiccation, swabs moistened with saline were placed over the surgery incision sites. Body temperature was monitored by a rectal probe thermometer and maintained at 37 °C ± 0.3 °C by means of a homoeothermic blanket system (Harvard Apparatus). Upon the completion of surgery, MAP was allowed to stabilise for 15 min. Blood was then withdrawn (up to 1 mL/min into heparinised syringes containing 100 IU/mL heparin mixed with normal saline) through the cannula inserted in the carotid artery in order to achieve a fall in MAP to 35 ± 5 mmHg, which was recorded with a pressure transducer (attached to the femoral artery cannula, 844-31 Memscap, Durham, USA) and coupled to a PowerLab 8/30 data acquisition system (AD Instruments Pty Ltd., Castle Hill, Australia). Thereafter, MAP was maintained at 35 ± 5 mmHg for a period of 90 min either by further withdrawal of blood during the compensation phase or administration of the shed blood during the decompensation phase. At 90 min after initiation of haemorrhage (or when 25 % of the shed blood had to be re-injected to sustain MAP at 35 ± 5 mmHg), resuscitation through the jugular vein was performed with the remaining shed blood (mixed with 100 IU/mL heparinised saline) over a period of 5 min plus a volume of Ringer's lactate identical to the volume of shed blood. Treatment or vehicle was also administered intravenously. One hour after resuscitation, an infusion of Ringer's lactate (1.5 mL/kg/h) was started as fluid replacement and was maintained throughout the experiment for a total of 3 h. During the final 3 h, urine was collected through a catheter placed in the bladder to estimate the creatinine clearance. Under deep anaesthesia, the heart was removed to terminate the experiment 4 h after resuscitation. Sham-operated rats were used as control and underwent identical surgical procedures, but without haemorrhage or resuscitation.

Rats were treated with either ISO-1 (25 mg/kg) or its vehicle (5 % DMSO + 95 % Ringer's lactate). An initial bolus of ISO-1 treatment (10 mg/kg) or vehicle was administered intravenously immediately after resuscitation. 30 min after resuscitation, an infusion of ISO-1 (3.75 mg/kg/h, i.v.) or vehicle was started and maintained throughout the experiment for a total of 4 h.

Sample Collection - Acute Haemorrhagic Shock Model (Short-term Follow-up)

Rats remained anaesthetised with sodium thiopentone (120 mg/kg i.p.) before culling by Schedule 1 killing. Up to 8 mL of blood was taken from the right ventricle of the heart via cardiac puncture into non-heparinised 5 mL syringes and immediately decanted into 1.1 mL serum gel tubes (Sarstedt, Germany). The blood was centrifuged (10,000 g for 5 min) to obtain the serum, which was

subsequently stored at -80 °C until analysis. Organs (heart, lungs, liver and kidneys) were excised of which one section was snap frozen in liquid nitrogen and stored at -80 °C, and another section was placed in 10 % formalin for 24-48 h; followed by transfer to 70 % ethanol until further analysis. All organ injury/dysfunction parameters (urea, creatinine, alanine aminotransferase [ALT], aspartate aminotransferase [AST], creatine kinase [CK], amylase and lactate dehydrogenase [LDH]) in the serum were measured in a blinded fashion by a clinical pathology diagnostic laboratory (MRC Harwell Institute, Oxfordshire, UK).

Acute Haemorrhagic Shock Model (Long-term Follow-up)

Thirty rats were randomised into three groups: Sham + vehicle (n = 6); HS + vehicle (n = 12); HS + ISO-1 (25 mg/kg; n = 12). At 15 min prior to anaesthesia, analgesia with tramadol (10 mg/kg i.p.) was administered. Rats were then anaesthetised with ketamine-xylazine (ketamine, 100 mg/kg; xylazine, 10 mg/kg i.m.) and the pedal reflex was tested to ensure adequate anaesthesia. Cannulation with polyethylene catheters of the left femoral artery and left femoral vein was performed. Body temperature was monitored by a digital ear thermometer and maintained at 36.5 °C ± 0.5 °C by means of a homoeothermic blanket system (Harvard Apparatus). Upon completion of surgery, MAP was allowed to stabilise for 15 min. Blood was then withdrawn (up to 1 mL/min into heparinised syringes containing 100 IU/mL heparin mixed with normal saline) through the cannula inserted in the femoral artery in order to achieve a fall in MAP to 40 ± 2 mmHg, which was recorded with a pressure transducer (attached to the femoral artery cannula) and coupled to a PowerLab 8/30 (AD Instruments Pty Ltd., Castle Hill, Australia). Thereafter, MAP was maintained at 40 ± 2 mmHg for a period of 90 min either by further withdrawal of blood or administration of the shed blood. At 90 min after initiation of haemorrhage (or when 25 % of the shed blood had to be re-injected to sustain MAP at 40 ± 2 mmHg), resuscitation through the femoral vein was performed with the remaining shed blood over a period of 5 min plus 1.5 mL/kg Ringer's lactate (i.v.). An initial bolus of ISO-1 treatment (12.5 mg/kg) or vehicle was then administered intraperitoneally. Sham-operated rats were used as control and underwent identical surgical procedures, but without haemorrhage or resuscitation. At 20 min after resuscitation was completed, the catheters were removed, the femoral vessels were ligated, and the incision was closed with sutures. Rats were allowed to recover from the anaesthesia and 12 h later given tramadol (5 mg/kg i.p.) and a second dose of ISO-1 (12.5 mg/kg) or vehicle. At 24 h post-resuscitation, rats were anaesthetised with ketamine-xylazine (100 mg/kg ketamine, 10 mg/kg xylazine i.m.) and samples were collected. Cannulation of the left carotid artery with a polyethylene catheter was performed to measure the mean arterial pressure (MAP) and heart rate (HR); after which up to 5 mL blood was taken into non-heparinised blood collection tubes. The blood was centrifuged (10,000 g for 5 min) to obtain the serum and stored at -80 °C until analysis. Organ collection and measurement of organ injury/dysfunction parameters (Hospital Universitário Professor Polydoro Ernani de São Thiago, Brazil) was performed as described in the short-term follow-up acute model.

Western Blot Analysis

Semi-quantitative immunoblot analysis was carried out in kidney and liver tissue samples as previously described (1). Briefly, kidney and liver samples from the short-term follow-up acute HS model were homogenised in buffer and centrifuged (1320 g, 5 min, 4 °C). To obtain the cytosolic fraction,

supernatants were centrifuged (16,125 g, 4 °C, 40 min). The pelleted nucleoli were resuspended in extraction buffer and centrifuged (16,125 g, 20 min, 4 °C). Protein content was determined on both nuclear and cytosolic extracts using bicinchoninic acid (BCA) protein assay (Thermo Fisher Scientific Inc, Rockford, IL). Proteins were separated by 8 % sodium dodecyl sulphate polyacrylamide (SDS-PAGE) gel electrophoresis and electrotransferred to polyvinylidene difluoride (PVDF) membrane. After blocking (1 h in 10 % dry milk solution), membranes were incubated with primary antibodies in 5 % blocking solution overnight [1:1000 rabbit anti-NF-κB, 1:1000 rabbit anti-IKKβ, 1:1000 rabbit anti-Ser$^{176/180}$ IKKα/β (from Cell Signaling), 1:1000 rabbit anti-NLRP3 inflammasome (from Abcam), 1:1000 mouse anti-caspase 1 (p20) (from Adipogen)] followed by incubation with appropriate HRP-conjugated secondary antibodies. Proteins were detected with an ECL detection system and quantified by densitometry using analytic software (Quantity-One; Bio-Rad, Hercules, CA). Results were normalised with respect to densitometric values of tubulin for cytosolic proteins or histone H3 for nuclear proteins.

CD68 Immunohistochemical Staining

Lung tissue was collected at the end of the experiment and kept in formalin for 24-48 h at room temperature, before it was transferred to 70 % ethanol. The tissue was embedded in paraffin and sectioned. The slides were then deparaffinised and rehydrated with xylene and ascending concentrations of ethanol. The next step included an antigen retrieval to unmask unspecific antigen bindings followed by incubation with rabbit anti-CD68 antibody ED1 (1:2000 in TBS with 1 % BSA + 10 % rabbit serum; catalogue no. MCA341R; AbD Serotec) for 16 h at 4 °C. The sections were then incubated with labelled polymer-HRP antibody (ab236469 - Rabbit specific HRP/DAB Detection IHC Detection Kit - Micro-polymer) for 15 min. The slides were counterstained with Harris haematoxylin solution, dehydrated and mounted on slides. Images were developed using a NanoZoomer Digital Pathology Scanner (Hamamatsu Photonics K.K. Japan). NDP Viewer software was used for image analysis. CD68 positive cells were counted in 10 randomly selected fields (300 µm) in a double-blinded manner by two independent investigators.

Quantification of Myeloperoxidase Activity

Determination of myeloperoxidase activity in lung and liver tissue samples was performed as previously described (1). Lung and liver tissue samples from the long-term follow-up acute HS model were homogenised in liquid nitrogen with a pestle and mortar and then homogenised in 80 mM phosphate-buffered saline (PBS), pH 5.4, containing 0.5 % hexadecyltrimethylammonium bromide. The homogenate was then centrifuged at 13,000 g at 4 °C for 10 min and the supernatant was assayed for myeloperoxidase (MPO) activity by measuring the H_2O_2-dependent oxidation of 3,3,5,5-tetramethylbenzidine (TMB). MPO activity was determined colorimetrically using an ultra-microplate reader (EL 808, BioTek Instruments, INC, USA) set to measure absorbance at 650 nm. Total protein content in the homogenate was estimated using the BCA assay (Thermo Fisher Scientific, Rockford, IL), according to the manufacturer's instructions. MPO activity was expressed as optical density at 650 nm per mg of protein.

Supplementary Tables

Supplementary Table 1: ELISA

<table>
<tr><th></th><th>Human MIF ELISA</th><th>Rat MIF ELISA</th></tr>
<tr><td>Intra-assay variability</td><td>Not reported by manufacturer</td><td>CV%<8%</td></tr>
<tr><td>Inter-assay variability</td><td>Not reported by manufacturer</td><td>CV%<10%</td></tr>
<tr><td>Lower limits of detection</td><td>31.3 pg/mL</td><td>62.5 pg/mL</td></tr>
<tr><td>Linearity-of-dilution assessment</td><td>Not reported by manufacturer</td><td>To assess the linearity of the assay, samples were spiked with high concentrations of rat MIF in various matrices and diluted with the Sample Diluent to produce samples with values within the dynamic range of the assay.
<table>
<tr><th></th><th>Sample</th><th>Serum (n=4)</th></tr>
<tr><td>1:5</td><td>Average %</td><td>95</td></tr>
<tr><td></td><td>Range %</td><td>90-100</td></tr>
<tr><td>1:10</td><td>Average %</td><td>98</td></tr>
<tr><td></td><td>Range %</td><td>92-102</td></tr>
<tr><td>1:20</td><td>Average %</td><td>94</td></tr>
<tr><td></td><td>Range %</td><td>89-99</td></tr>
<tr><td>1:40</td><td>Average %</td><td>92</td></tr>
<tr><td></td><td>Range %</td><td>82-96</td></tr>
</table>
</td></tr>
</table>

Supplementary Table 2: Antibodies

Antibody	Source	Catalogue number
Rabbit anti-NF-κB	Abcam	ab86299
Rabbit anti-IKKα/β	Abcam	ab178870
Rabbit anti-$Ser^{176/180}$ IKKα/β	Abcam	ab17943
Rabbit anti NLRP3 inflammasome	Abcam	ab214185
Mouse anti-caspase 1 (p20)	Adipogen	AG-20B-0042-C100
Rabbit anti-CD68 antibody ED1	AbD Serotec	MCA341R
Rabbit specific HRP/DAB Detection IHC Detection Kit - Micro-polymer	Abcam	ab236469

Supplementary Figures

Figure legends

Supplementary Figure 1: Schematic representation of the acute HS models. The experimental procedures at each stage of the **(A)** short-term follow-up and **(B)** long-term follow-up acute HS model are shown.

Supplemental Figure 2: MIF gene expression is elevated in trauma patients. Original data was obtained from the Gene Expression Omnibus under dataset accession number GSE36809 which was published by (2). RNA was extracted from whole blood leukocytes over a 28-day time course from trauma patients (n = 167) and matched healthy controls (n = 37). Data were reanalysed for MIF gene expression. Data are expressed as mean ± SEM. Statistical analysis was performed using one-way ANOVA followed by a Bonferroni's *post-hoc* test. *$p<0.05$ denoted statistical significance.

Supplemental Figure 3: MIF gene expression does not differ between uncomplicated and complicated recovery patient groups. Original data was obtained from the Gene Expression Omnibus under dataset accession number GSE36809 which was published by Xiao and colleagues (2). Data were reanalysed for MIF gene expression in uncomplicated (n = 55) and complicated (n = 41) recovery patient groups. Data are expressed as mean ± SEM. Statistical analysis was performed using two-way ANOVA followed by a Bonferroni's *post-hoc* test.

Supplemental Figure 4: Effect of ISO-1 treatment on pulmonary $CD68^+$ cell infiltration in a short-term follow-up acute HS model. (**A**) Representative images of immunostained lungs are presented. (**B**) $CD68^+$ cells per field in the lungs of vehicle and ISO-1 treated rats. Data are expressed as median with range of 6-7 animals per group. Statistical analysis was performed using one-way ANOVA followed by a Bonferroni's *post-hoc* test. *$p<0.05$ denoted statistical significance.

Supplementary Figure 5: Serum MIF levels are strongly associated with clinical chemistry and MAP in a short-term follow-up acute HS model. Heatmap of the correlation between MIF (serum levels 4 h post-resuscitation) and serum clinical chemistry parameters (urea; creatinine; ALT, alanine transaminase; AST, aspartate transaminase; amylase; CK, creatine kinase; LDH, lactate

dehydrogenase) and mean arterial pressure (MAP). A colour closer to red indicates a negative correlation, blue indicates a positive correlation and white indicates no correlation.

References

1. Patel NM, Oliveira FRMB, Ramos HP, Aimaretti E, Alves GF, Coldewey SM, et al. Inhibition of Bruton's Tyrosine Kinase Activity Attenuates Hemorrhagic Shock-Induced Multiple Organ Dysfunction in Rats. Annals of Surgery. 2021 Dec 27;

2. Xiao W, Mindrinos MN, Seok J, Cuschieri J, Cuenca AG, Gao H, et al. A genomic storm in critically injured humans. The Journal of Experimental Medicine [Internet]. 2011 Dec 1 [cited 2021 Aug 6];208(13):2581. Available from: /pmc/articles/PMC3244029/
