# Supplementary figures and images for: Inhibition of Macrophage Migration Inhibitory Factor Activity Attenuates Haemorrhagic Shock-Induced Multiple Organ Dysfunction in Rats
Source: Front Immunol. 2022 Apr 6;13:886421. doi: 10.3389/fimmu.2022.886421 (PMC9019168; doi:10.3389/fimmu.2022.886421)

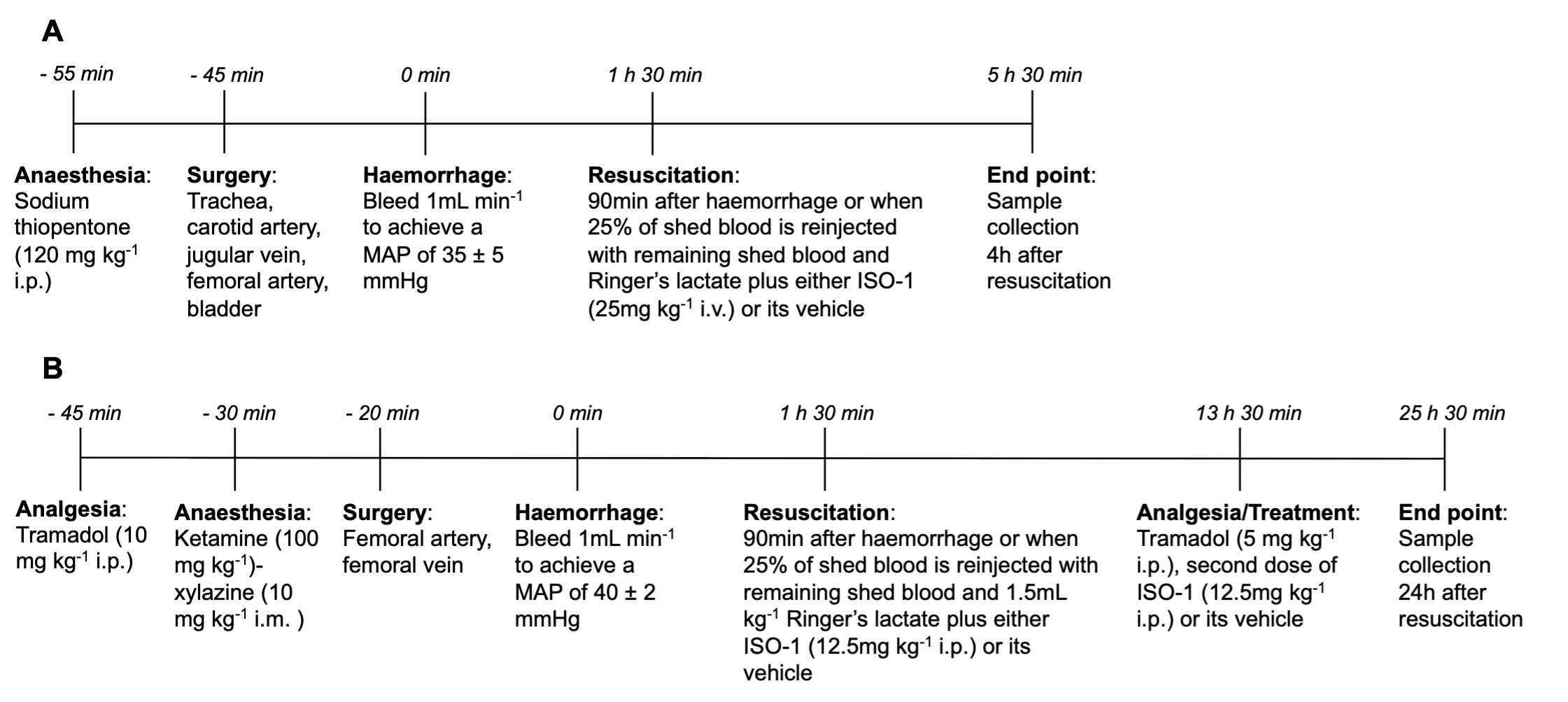

Supplement: Supplementary file 2 [file Image_1.jpeg]

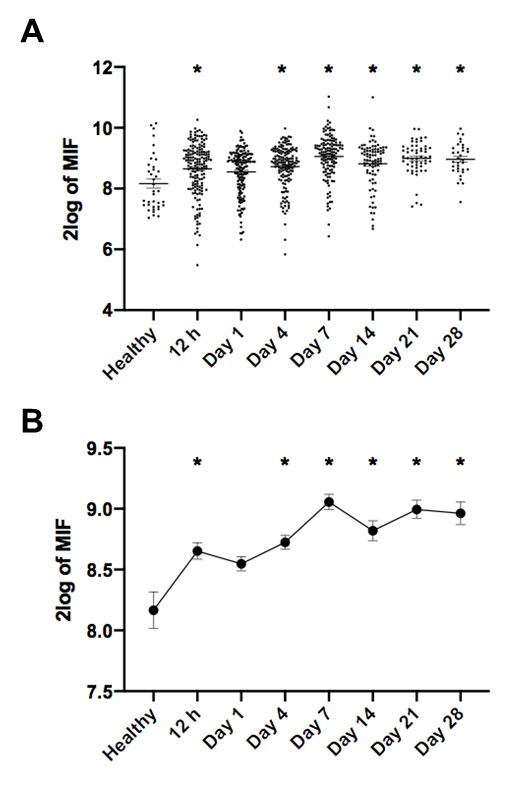

Supplement: Supplementary file 3 [file Image_2.jpeg]

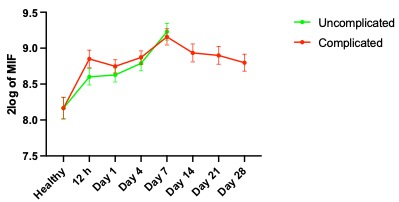

Supplement: Supplementary file 4 [file Image_3.jpeg]

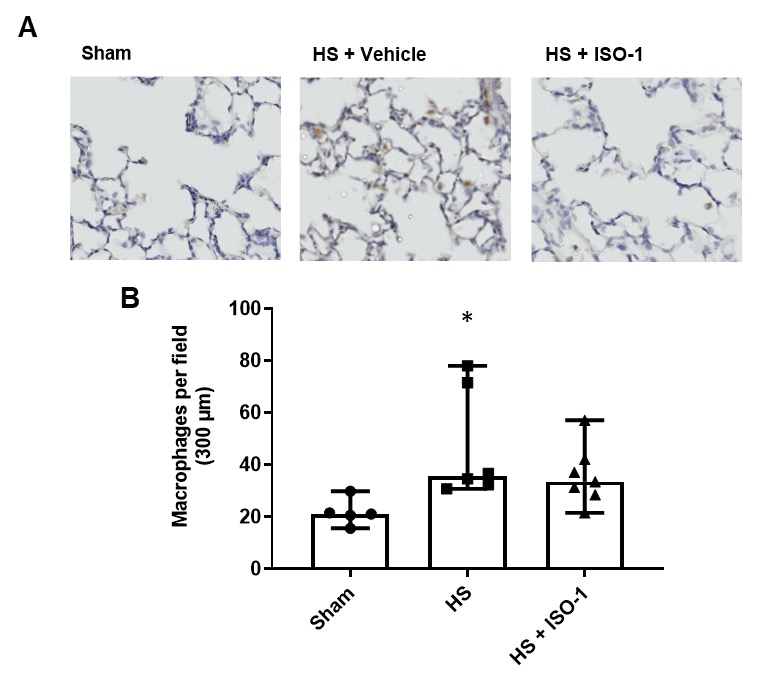

Supplement: Supplementary file 5 [file Image_4.jpeg]

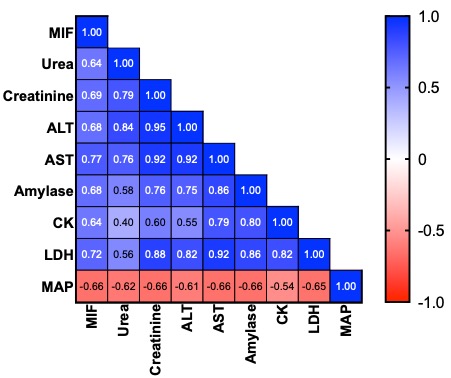

Supplement: Supplementary file 6 [file Image_5.jpeg]
